# Hearing Dysfunction in *Xpa*-Deficient Mice

**DOI:** 10.3389/fnagi.2017.00019

**Published:** 2017-02-10

**Authors:** Hitomi Shinomiya, Daisuke Yamashita, Takeshi Fujita, Eiji Nakano, Go Inokuchi, Shingo Hasegawa, Naoki Otsuki, Chikako Nishigori, Ken-ichi Nibu

**Affiliations:** ^1^Department of Otolaryngology-Head and Neck Surgery, Kobe University Graduate School of MedicineKobe, Japan; ^2^Division of Dermatology, Department of Internal Related Graduate School of Medicine, Kobe UniversityKobe, Japan

**Keywords:** XP, SNHL, spiral ganglion neuron, ABR, organ of Corti, stria vascularis

## Abstract

Xeroderma pigmentosum (XP) is a rare recessive heredity disease caused by DNA repair impairment characterized by photosensitivity and neurologic symptoms in half of the cases. There are eight subtypes of XP: XP-A–XP-G and XP variant. Among eight subtypes, XP complementation group A (XP-A) display the lowest DNA repair ability and the severest cutaneous and neurologic symptoms. While its pathogenesis of skin symptoms have been well-studied, that of neurological symptoms, including sensorineural hearing loss (SNHL) remains unknown. Basic studies have suggested that SNHL may be caused by inner ear damage, including damage to the spiral ganglion neurons and organ of Corti, and that the XP-A is associated with most severe form of SNHL in humans. Here, we report the occurrence of SNHL in *Xpa*-deficient mice. *Xpa*-deficient mice and wild-type mice underwent measurements for auditory brainstem response, and the results revealed that *Xpa*-deficient mice exhibited significantly greater (*p* < 0.01) ABR thresholds at 4, 8, and 16 kHz than the wild-type mice. Furthermore, the number of spiral ganglion neurons was reduced in *Xpa*-deficient mice compared with that in wild-type mice, indicating that hearing loss may be related to spiral ganglion neuron deficiency, consistent with the few reports published in human patients with XP. These results provide important insights into the pathogenesis of SNHL in patients with XP-A.

## Introduction

Xeroderma pigmentosum (XP) is an autosomal recessive hereditary disease characterized by increased susceptibility to freckle-like pigmentation and skin cancers at sun-exposed body sites (DiGiovanna and Kraemer, 2012). Furthermore, some patients display neurological manifestations, including hearing impairment. XP is classified into seven genetic complementation groups deficient in nucleotide excision repair (A through G) and an XP-variant type; the relative frequency and severity of cutaneous and neurological symptoms differ depending on the subtype. XP occurs at a higher frequency in Japan (1:22,000; Hirai et al., 2006) than in the United States (1:250,000; Robbins et al., 1974). Approximately 50% of all Japanese patients with XP are assigned to the XP complementation group A (XP-A), and most patients with XP-A exhibit severe neurological manifestations (Nishigori et al., 1994).

During daily life, humans are exposed to various genotoxic hazards; in order to protect the body from these hazards, its constituent cells have multiple pathways for repair of DNA damage caused by environmental agents, including endogenous factors such as reactive oxygen species (ROS) and reactive nitrogen species (RNS), and exogenous factors such as UV radiation (UVR), ionizing radiation, electrophilic chemical adducts, and certain drugs. If the DNA repair pathway fails to function properly, replication, and transcription errors occur, resulting in cell death and mutation. The occurrence of mutations leads to failure of normal metabolism, resulting in aging and carcinogenesis (Menck and Munford, 2014). One of the major DNA repair pathways is nucleotide excision repair (NER), in which XPA–XPG proteins function in a complementary manner (Naegeli and Sugasawa, 2011). However, patients with XP lack one of the NER protein complexes, and therefore show an impaired capacity to properly repair DNA lesions that cause structural distortion, such as dipyrimidine photoproducts, which is caused by ultraviolet radiation (UVR), as well as DNA adducts caused by drugs (Cleaver, 2005). In addition, several studies have shown that NER plays a partial role in repair of damage caused by ROS produced during endogenous metabolism (Menck and Munford, 2014).

The skin and mucosal symptoms in patients with XP are attributed to deficiencies in the repair of UVR-induced DNA damage. Patients with XP-A suffer severe sunburn upon minimum sun exposure and gradually develop many freckle-like pigmentated maculae. These individuals also develop skin cancers such as basal cell carcinoma, squamous cell carcinoma, and malignant melanoma, in addition to actinic keratosis, which is a precancerous lesion. Unless photoprotection is adhered to, most patients with XP-A develop skin cancer before the age of 10. Moreover, most patients with XP-A also exhibit progressive degenerative neurological manifestations, including mental deterioration, microcephaly, sensorineural hearing loss (SNHL), abnormal speech, areflexia, and peripheral neuropathy (Nishigori et al., 1994; Bradford et al., 2011). Hearing loss in XP-A patients may appear from the age of about 6 years and up through their 20s, eventually becoming severe in their late 30s–40s (Nishigori et al., 1994; Totonchy et al., 2013). The representative manifestation of hearing impairment in patients with XP-A harboring a homozygous IVS3-1G>C missplice mutation of *XPA* is bilateral severe to profound hearing loss; the audiogram is usually horizontal or worse at higher frequencies.

However, the mechanisms mediating these neurological symptoms in patients with XP-A remain unclear because UVR cannot penetrate through the skin layers to reach the nervous system. Some studies have shown that generation of ROS results in neuronal cell death in patients with XP (Anttinen et al., 2008; Viana et al., 2013). Additionally, researchers have outlined two major hypotheses to explain the pathogenesis of neurological degeneration in XP: (1) there is a deficiency in the ability to repair cyclo-purine, which is induced by oxidative stress through daily aerobic metabolism; or (2) cells cannot effectively repair some types of ROS generated during normal metabolism. However, the specific mechanisms remain unknown.

Among the neurological symptoms observed in patients with XP-A, hearing impairment has been shown to develop at a relatively young age (Nishigori et al., 1994). However, because XP is a very rare disorder, few studies have examined the progression of SNHL. Therefore, the present study was conducted to characterize the mechanisms of neurological dysfunction and hearing impairment in patients with XP through evaluation of hearing loss in *Xpa*-deficient mice and the mechanisms involved.

## Materials and methods

### Animals

*Xpa*-deficient mice with CBA (15), C57BL/6, and CD-1 chimeric backgrounds, which were first generated by insertion of neomycin cassette sequences into exon 4 of the *Xpa* gene using embryonic stem (ES) cell techniques (Nakane et al., 1995), were backcrossed with hairless albino mice from the Balb/cA Kud-hr inbred strain (Itoh et al., 2005). The resulting hairless albino *Xpa* (+/–) mice were kindly provided by Professor Kiyoji Tanaka (Nakane et al., 1995). We repeatedly mated these mice, and the resulting hairless albino *Xpa* (+/+) mice were used as wild-type (WT) mice whereas hairless-albino-*Xpa* (–/–) mice were used as *Xpa*-deficient mice. Mice aged 38–40 weeks were used in this study. Six *Xpa*-deficient mice were used for evaluation of hearing level, spiral ganglion neuron (SGN) count, stria vascularis thickness, and hair cell count, and five WT mice served as the control group. Mice were housed under specific pathogen-free conditions with a 12-h light/dark cycle (lights on at 6:00 A.M.; lights off at 6:00 P.M.). All animal procedures were approved by the Institutional Review Board of Kobe University Graduate School of Medicine (Permit Number: P140613), and animal experiments were conducted according to the Guidelines of the Institutional Animal Care and Use Committee of Kobe University Graduate School of Medicine. All attempts were made to minimize animal use and suffering.

### Evaluation of hearing function by auditory brainstem response (ABR)

Mouse hearing function was determined in six *Xpa-*deficient and five WT mice by measuring the ABR at 38–40 weeks of age, as previously described (Fujita et al., 2015). Prior to the measurements, the mice were anesthetized by intraperitoneal injection of midazolam (10 mg/kg), medetomidine (37.5 μg/kg), and butorphanol tartrate (0.5 mg/kg). ABR was measured using waveform storing and stimulus control with Scope software incorporated in a PowerLab system (PowerLab2/26; AD Instruments, Castle Hill, Australia), and electroencephalogram (EEG) recording was performed with an extracellular amplifier AC PreAmplifier (P-55; Astro-Med, West Warwick, RI, USA). Sound stimuli were produced by a coupler-type speaker (ES1spc; Bio Research Center, Nagoya, Japan) inserted into the external auditory canal of mice. Tone burst stimuli, with a 0.2-ms rise/fall time (cosine gate) and 1-ms flat segment at frequencies of 4, 8, and 16 kHz were generated, and the amplitude was specified using a sound generator and an attenuation real-time processor and programmable attenuator (RP2.1 and PA5; Tucker-Davis Technologies, FL). Sound-level calibrations were performed using a sound level meter (NA-42; Rion, Tokyo, Japan). For recording, stainless steel needle electrodes were placed at the vertex and ventrolateral to the left and right ears. Generally, ABR waveforms were recorded for 12.8 ms at a sampling rate of 40,000 Hz using 50–5000 Hz band-pass filter settings; waveforms from 256 stimuli at a frequency of 9 Hz were averaged. In each animal, ABR was measured in both ears. ABR waveforms were recorded at sound pressure level (SPL) intervals of 5 dB down from the maximum amplitude until no waveform could be visualized. The lowest sound pressure level for which the last waveform was detected was recorded as the animal's hearing level. If a strong waveform suddenly disappeared when the sound level was lowered by 5 dB, we considered the hearing level to be the interval between the sound pressure levels. The largest wavelet was used for determination of ABR threshold.

### Morphological evaluation of cochleae

After ABR measurements, the mice were sacrificed by deep anesthesia and cervical dislocation, and cochleae were removed. Removed cochleae were then fixed in 4% paraformaldehyde overnight, incubated in 10% ethylenediaminetetraacetic acid disodium salt dehydrate (pH 7.0, Muto Pure Chemicals, Tokyo, Japan) at room temperature for 2 days. Cochleae were dehydrated through a graded ethanol series and xylene, embedded in paraffin, and then sectioned at 3.0 μm in the midmodiolar plane.

Cochlear tissue sections on slides were stained with hematoxylin (Muto Pure Chemicals, Tokyo, Japan) and eosin (Wako Pure Chemicals Industries, Osaka, Japan), referred to as H-E staining, for histopathological evaluations. Cochlear specimens were observed using a light microscope system (BZ-9000; Keyence, Osaka, Japan) and the digital images were saved.

### Spiral ganglion neuron (SGN) count

SGNs were counted using six *Xpa*-deficient and five WT mice. Three midmodiolar sections (30 μm apart) were selected from each cochlea. The area of Rosenthal's canal and the cochlear turns were quantified by measuring their cut surfaces using BZ-H1AE microscope analysis software (Keyence, Osaka, Japan). All SGNs within each measured area were counted for each cochlear turn (apical, middle, basal). The SGN cell density was determined as the average number of nuclei per 10,000 μm^2^ of Rosenthal's canal for three sections in each cochlear turn, as described previously (Fujita et al., 2012).

### Measurement of stria vascularis thickness

Stria vascularis thickness was measured using six *Xpa*-deficient and five WT mice. The slides were evaluated from × 40 images. The thickness was defined as the distance between the margin of the stria and the junction of the basal cells with the spiral ligament halfway between the attachment of Reissner's membrane and the spiral prominence (Han et al., 2016a). Six to seven sections of the apical, middle, and basal regions of the cochlea for each mouse and the averages for each region were calculated.

### Hair cell count

Hair cells were counted using six *Xpa*-deficient and five WT mice. Outer hair cells (OHCs) and inner hair cells (IHCs) were counted in × 40 images of the apical, middle, and basal cochlear regions. Six to thirteen sections of each turn were evaluated in one cochlea per mouse. Hair cells were identified by the presence of a nucleus. Percentage of OHC survival was calculated as the number of intact OHCs present among the three OHCs that should be observed in cochlear sections of mice with normal hearing. Percentage of IHC survival was calculated as the number of intact IHCs present among the one IHC that should be observed in cochlear sections of mice with normal hearing (Han et al., 2016b).

### Data analysis

All data are reported as the mean ± standard error (*SE*). The ABR threshold levels, SGN density, stria vascularis thickness, and hair cell percentage were assessed by non-paired *t*-tests (R software ver. 3.0.2; R Foundation for Statistical Computing, 2013). Differences with *p*-values of < 0.01 were considered significant.

## Results

### ABR thresholds in *Xpa*-deficient mice and WT mice

Our preliminary data suggested that *Xpa*-deficient mice had almost the same ABR thresholds as WT mice at 20 weeks of age (20–40 dB). At 60 weeks of age, the ABR thresholds of both *Xpa*-deficient and WT mice had nearly reached the upper limit of detection (i.e., for this ABR technique, 85 dB SPL at 4 kHz, 80 dB SPL at 8 kHz, and 80 dB SPL at 16 kHz; data not shown). Therefore, we used mice at 38–40 weeks of age in order to detect differences in ABR thresholds between the two genotypes.

**Figure 1:**
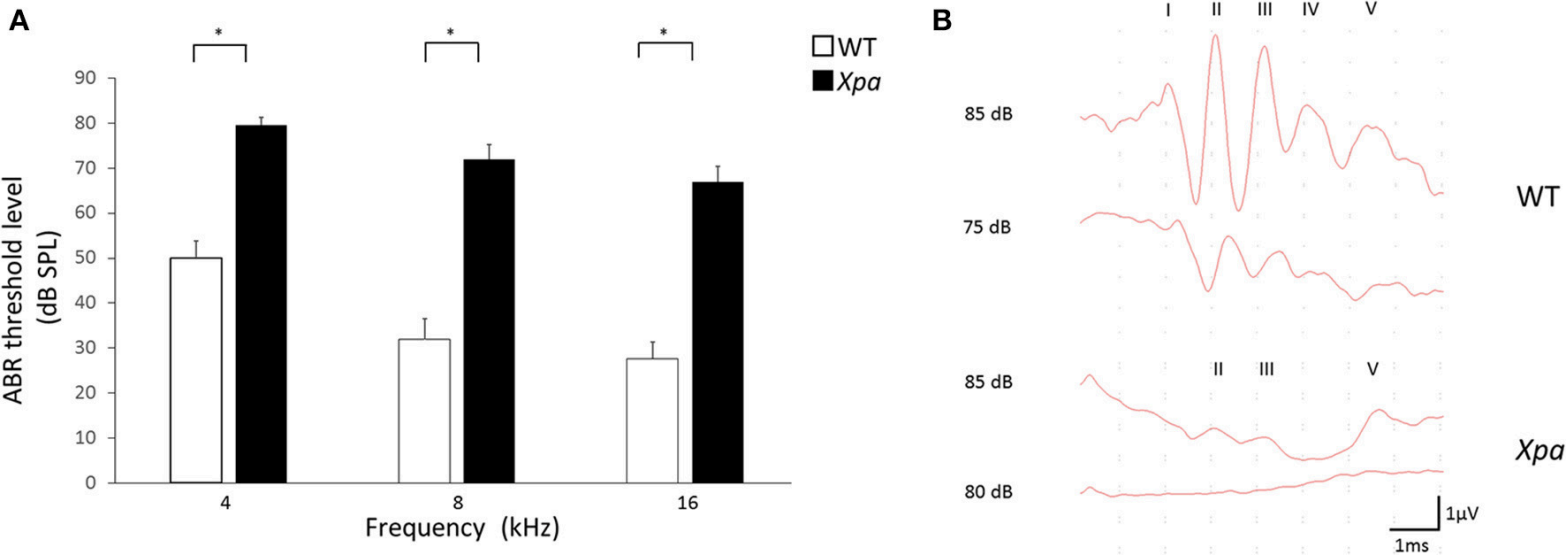
**(A) ABR threshold level in WT mice and ***Xpa***-deficient mice at frequencies of 4, 8, and 16 kHz**. Data are means ± *SE*. ^*^P < 0.01 for comparisons between WT mice and *Xpa*-deficient mice. **(B) Representative ABR waveforms at 4000 Hz in WT mice and *Xpa*-deficient mice**. ABR threshould of the *Xpa*-deficient mice was determined as 85 dB SPL.

Interestingly, *Xpa*-deficient mice exhibited significantly higher threshold elevation than WT mice at all ABR frequencies (4, 8, and 16 kHz; Figure 1A). The average threshold levels (±*SE*) in *Xpa*-deficient mice were 79.5 ± 1.7, 71.9 ± 3.4, and 67 ± 3.4 dB SPL at 4, 8, and 16 kHz, respectively, whereas those in WT mice were 50 ± 3.8, 31.9 ± 4.6, and 27.7 ± 3.7 dB SPL, respectively. Figure 1B shows the ABR waveforms of WT and *Xpa*-deficient mice.

### Morphological evaluation of the cochlea

**Figure 2:**
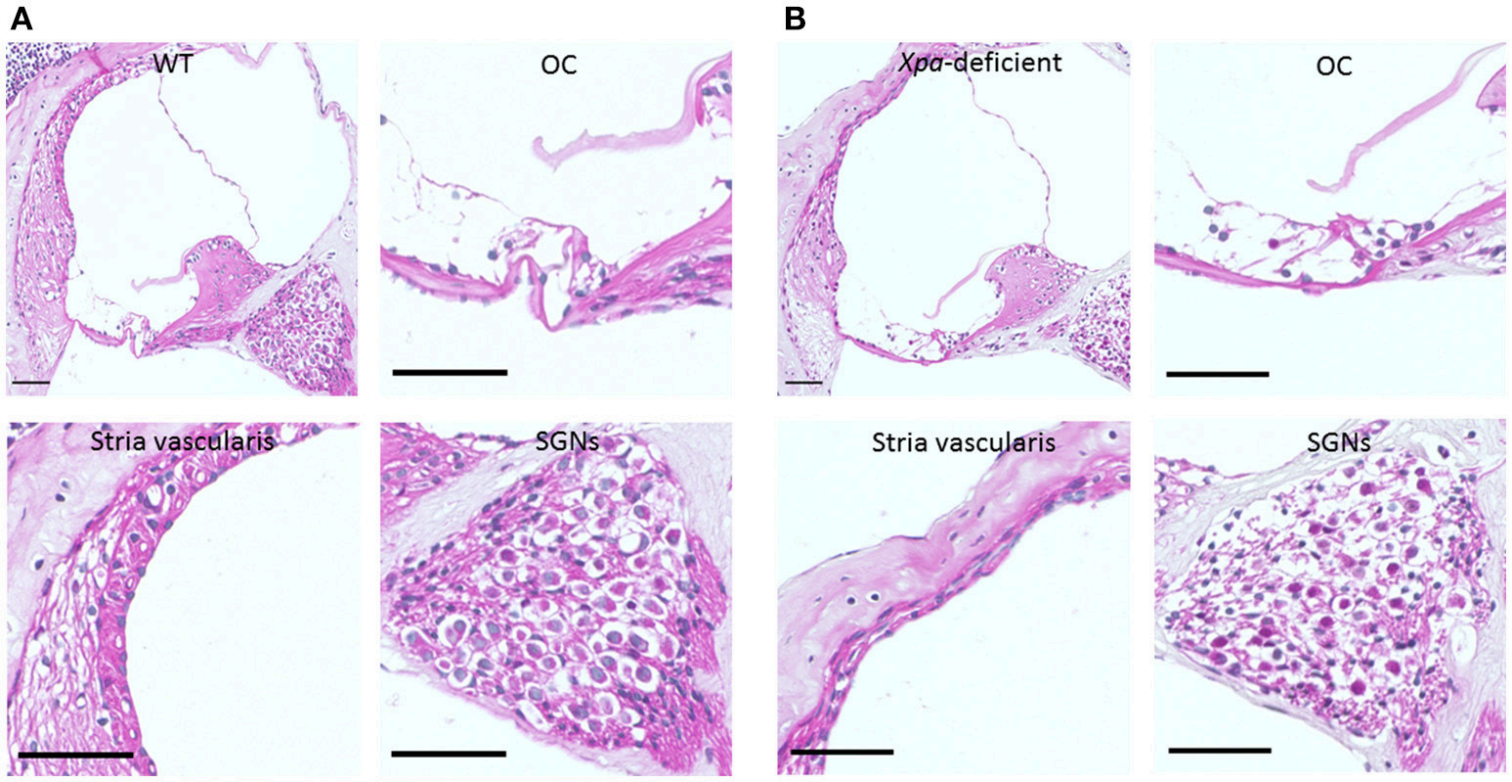
**Histopathology of the cochlear tissue of WT mice (A) and ***Xpa-***deficient mice (B)**. H-E staining; (scale bar = 50 μm). Cross-section through the middle turn of the cochlea parallel to the modiolus, organ of Corti (OC), stria vascularis, and spiral ganglion neurons (SGNs) in the middle turn of the cochlea.

Figure 2 show representative H-E sections of the cochlear middle turn in WT mice (A) and *Xpa*-deficient mice (B). There were no obvious inter-group differences in the stria vascularis or organ of Corti. However, the SGN showed lower density in *Xpa*-deficient mice than in WT mice.

### SGN count

**Figure 3:**
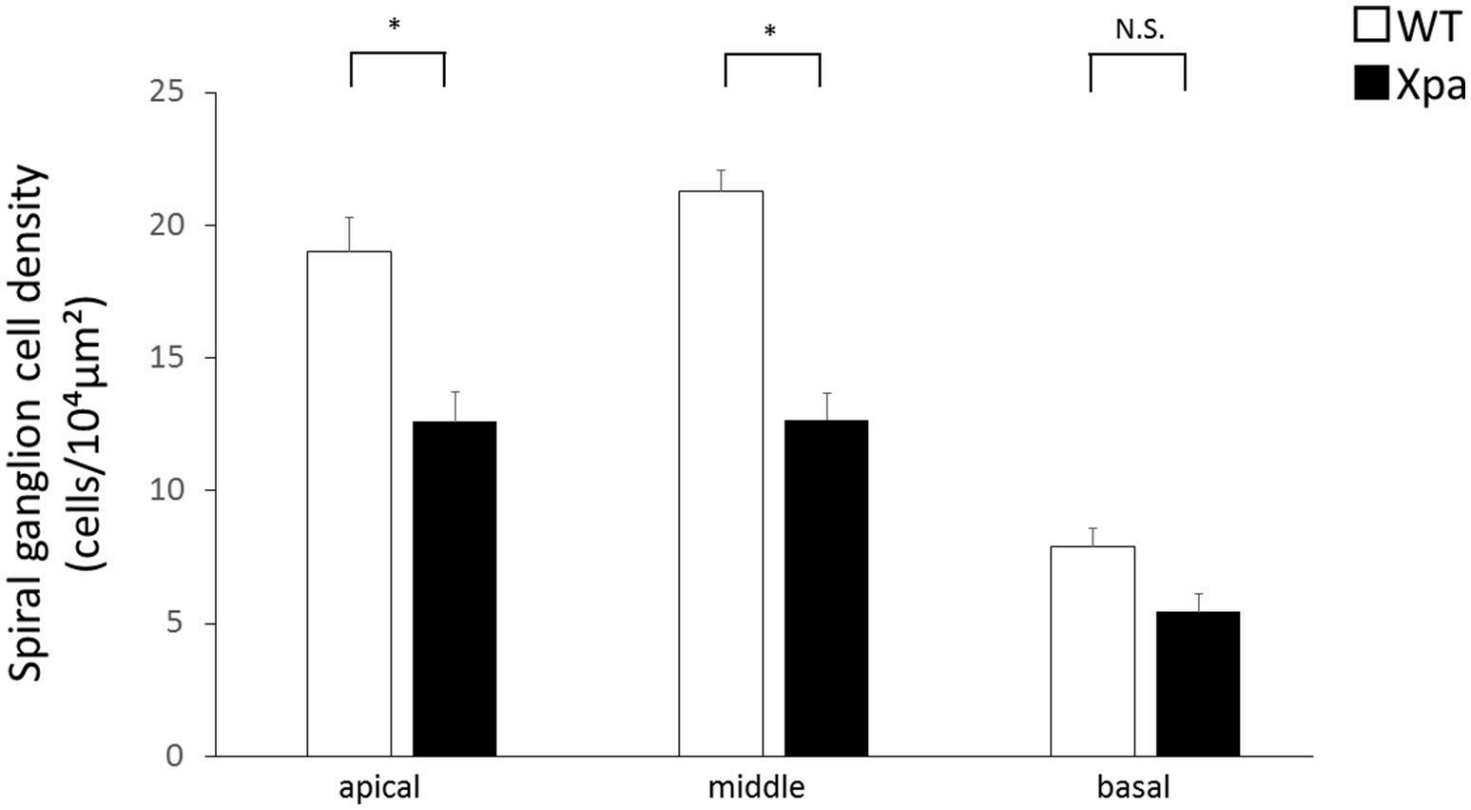
**Mean densities of spiral ganglion neurons in WT mice and ***Xpa***-deficient mice**. Data represent mean ± *SE*. ^*^P < 0.01 for comparisons between WT mice and *Xpa*-deficient mice. N.S., no significant difference between WT mice and *Xpa*-deficient mice.

In *Xpa*-deficient mice, the SGN count was lower in all regions of the cochlea relative to WT mice. The average SGN count (±*SE*) in *Xpa*-deficient mice and WT mice was 12.6 ± 1.1/10,000 and 19 ± 1.3/10,000 μm^2^, respectively, in the apical turn; 12.7 ± 1.0/10,000 and 21.3 ± 0.8/10,000 μm^2^, respectively, in the middle turn; and 5.4 ± 0.7/10,000 and 7.9 ± 0.7/10,000 μm^2^, respectively, in the basal turn (Figure 3). The SGN count was significantly lower in *Xpa*-deficient mice than in WT mice in the apical and middle turns (*p* < 0.01).

### Measurement of stria vascularis thickness

**Figure 4:**
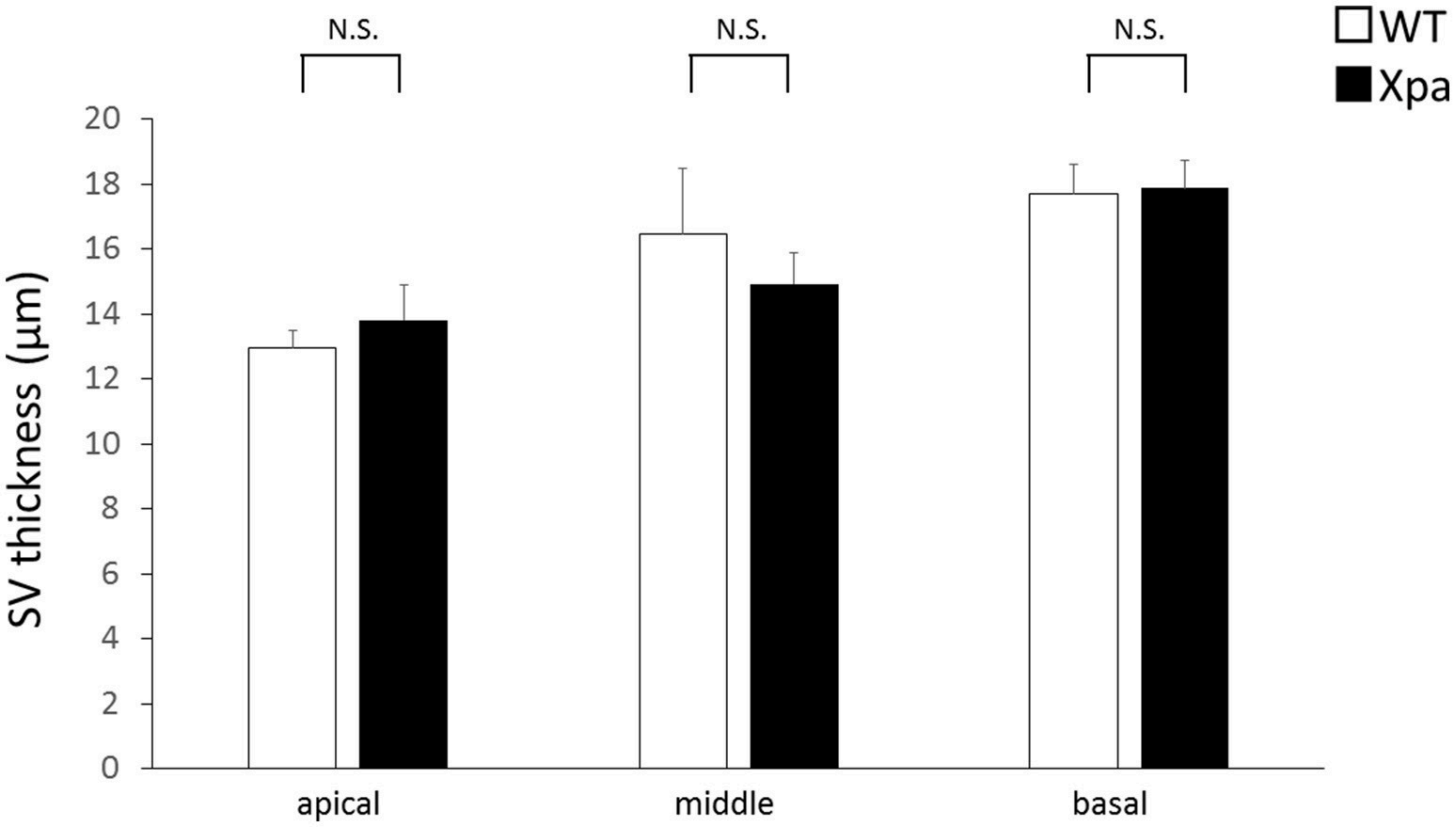
**Mean thickness of the stria vascularis (SV) in WT mice and ***Xpa***-deficient mice**. Data represent mean ± *SE*. N.S., no significant difference between WT mice and *Xpa*-deficient mice.

There was no significant difference in stria vascularis thickness between *Xpa*-deficient mice and WT mice (*p* > 0.01). The average thickness (±*SE*) of the stria vascularis in WT mice and *Xpa*-deficient mice group was 13.0 ± 0.5 and 13.8 ± 1.1 μm, respectively, in the apical turn; 16.5 ± 2.0 and 14.9 ± 1.0 μm, respectively, in the middle turn; and 17.7 ± 0.9 and 17.9 ± 0.9 μm, respectively, in the basal turn (Figure 4).

### Hair cell count

**Figure 5:**
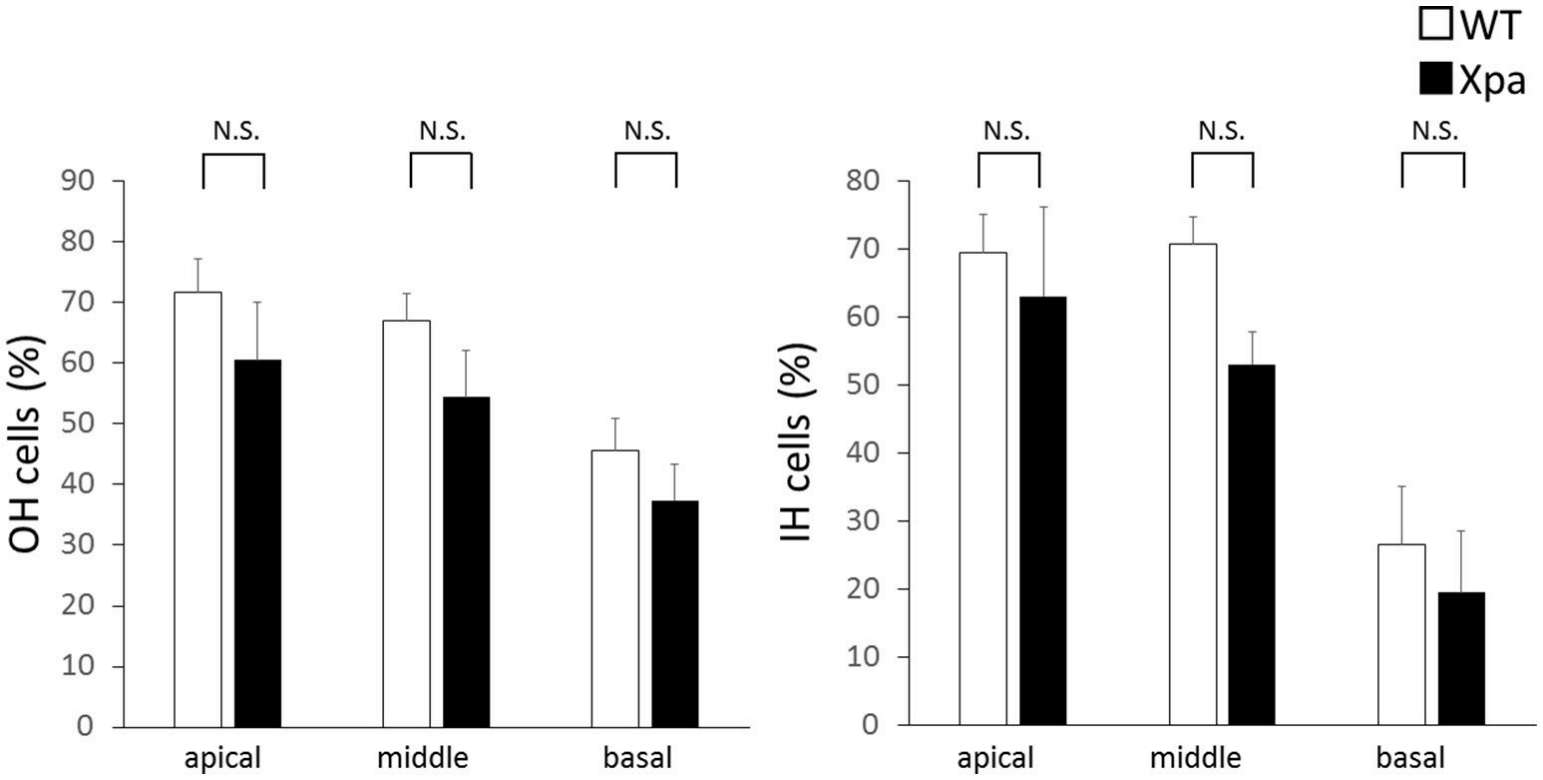
**Mean percentage of outer hair (OH) cells and inner hair (IH) cells remaining in WT mice and ***Xpa***-deficient mice**. Data are means ± *SE*. N.S., no significant difference between WT mice and *Xpa*-deficient mice.

There was no significant difference in the percentages of OHC and IHC remaining between *Xpa*-deficient mice and WT mice (*p* > 0.01). The average OHC percentage (±*SE*) in WT mice and *Xpa*-deficient mice was 71.7 ± 5.6 and 60.5 ± 9.5%, respectively, in the apical turn; 67.0 ± 4.6 and 54.4 ± 7.7%, respectively, in the middle turn; and 45.7 ± 5.3 and 37.4 ± 5.9%, respectively, in the basal turn. The average IHC percentage (±*SE*) in WT mice and *Xpa*-deficient mice was 69.5 ± 5.6 and 63.1 ± 13.0%, respectively, in the apical turn; 70.8 ± 3.9 and 53.1 ± 4.8%, respectively, in the middle turn; and 26.5 ± 8.5 and 19.4 ± 9.0%, respectively, in the basal turn (Figure 5).

## Discussion

In this study of hearing loss in *Xpa-*deficient mice, we found that these mice exhibited SNHL, with significantly higher hearing thresholds at frequencies of 4, 8, and 16 kHz, relative to those in WT mice. We also observed lower numbers of SGNs in these mice, providing important insights into the pathogenesis of SNHL in patients with XP-A. Unlike patients with XP-A, however, no other features suggestive of neurologic symptoms other than SNHL were evident in *Xpa*-deficient mice.

Very few studies have described the audiological characteristics of SNHL and otopathology in patients with XP. Kenyon et al. (1985) reported three XP patients who had impaired hearing and concluded that the hearing loss had a cochlear origin based on data for brainstem evoked potentials and electrocochleography (Kenyon et al., 1985). Additionally, Totonchy et al. (2013) found that 44 and 53% of patients with XP-A and XP-D, respectively, had hearing loss. Moreover, patients who exhibited hearing loss also tended to have other neurological manifestations (Totonchy et al., 2013). Similarly, Nishigori et al. (1994) reported that 15 of 29 patients with XP-A exhibited hearing impairment, most also having other neurological abnormalities such as peripheral nerve dysfunction, ataxia, mental retardation, and brain atrophy (Nishigori et al., 1994). It is known that there is a genotype-phenotype correlation in XP-A (Nishigori et al., 1994) and that more than 85% of Japanese patients with XP-A harbor a IVS-1G>C mutation, which results in absence of the XPA protein due to aberrant splicing, creating the severest clinical symptoms among XP-A patients. XP-A patients harboring IVS-1G>C begin to manifest neurologic abnormalities such as ataxia, hearing impairment, and neuroimaging abnormalities around 6 years of age (Nishigori et al., 1994; Ueda et al., 2012). It has also been reported that XP-A patients begin to manifest hearing impairment in their 20s, and that the condition becomes severe by their late 30s (Totonchy et al., 2013). This is in accord with our preliminary finding that the difference between the two mouse genotypes became apparent around 40 weeks of age, and is consistent with the notion that the neurologic symptoms of XP represent a progressive neurodegenerative disorder.

Few previous human studies have described the areas of the otological system responsible for hearing loss. In a recent autopsy study, researchers analyzed the temporal bone histopathology in a 44 year-old patient with XP-A who had suffered bilateral progressive SNHL that began during her second decade of life. Diffuse and severe atrophy of the organ of Corti and SGNs was evident. However, in the apical half of the cochlea, hair cells remained, despite severe degeneration of the SGNs, suggesting that the hearing loss had been partly due to neural degeneration (Totonchy et al., 2013; Viana et al., 2013). In another patient with XP who had had mild hearing loss and died of cancer at the age of 49 years, there were no abnormalities in the inner ear; however, the dorsal root ganglia showed neuronal loss. The author concluded that primary neuronal degeneration began initially in the peripheral nervous system (Robbins et al., 2002). In our present study of *Xpa*-deficient mice, SGNs were severely atrophic in comparison with those in age-matched controls, whereas there were no differences in stria vascularis thickness and the percentage of remaining hair cells between *Xpa*-deficient and WT mice.

The gene products responsible for each XP-complementation group are involved in each step of NER, including DNA damage recognition, DNA double-strand unwinding, excision of the damaged DNA with some flanking oligonucleotides, repair synthesis along with the daughter strand, and ligation of the newly synthesized fragment (Fousteri and Mullenders, 2008). Including XPA, the NER proteins exist in the cochleae of albino Fischer344 rats, and the NER pathway is involved in the repair of DNA damage caused by cisplatin. Additionally, cytoplasmic-to-nuclear translocation of XPA protein has been detected within SGNs during cycles of cisplatin treatment (Guthrie et al., 2008), indicating that XPA protein recognizes the DNA damage resulting from exogenous and/or endogenous hazards, and this is considered to be the main function of XPA protein in SGNs. Indeed, the effects of oxidative stress and the antioxidant system on the aging cochlea and central nervous system have been well-characterized. ROS, a natural byproduct of aerobic metabolism, causes chronic damage to ear structures (Staecker et al., 2001). Moreover, Brooks has reported that 8,5′-cyclopurine-2′-deoxynucleosides, which are induced by oxidative stress, may represent a candidate for production of the neurodegenerative DNA lesions in patients with XP (Brooks, 2008). We hypothesize that ROS generated during normal metabolism may cause SGN death in patients with XP-A.

Although our data do not fully demonstrate that hearing deficiency in patients with XP-A is caused by disruption of spiral neurons, it will be essential to examine cochlear morphology in younger mice, including immunostaining for detection of apoptosis in cochlear sections. Moreover, it may be informative to examine various brain regions in *Xpa*-deficient mice. Elucidation of the specific mechanisms leading to hearing loss in these mice may provide important insights into XP-A in humans and facilitate our understanding of other neurological deficiencies in patients with XP. This model system could be useful for assessing methods to preserve hearing in XP-A patients, and might provide a tool for screening of therapeutic drugs for XP-A in future. Furthermore, it might also provide insights into mechanisms of hearing loss in the general population.

## Conclusion

*Xpa*-deficient mice tend to exhibit SNHL at an earlier age than WT mice, and morphological analyses of the cochlea in *Xpa*-deficient mice suggest degeneration of SGNs.

## Author contributions

HS was responsible for data acquisition and analysis, and drafting of the manuscript. DY was responsible for design/conception of the study, and data acquisition and analysis. TF was responsible for drafting the manuscript and critical revision of the manuscript for intellectual content. EN was responsible for data acquisition and analysis. GI was responsible for analysis and interpretation of the data. SH was responsible for the design/conception of the study. NO was responsible for critical revision of the manuscript for intellectual content. CN was responsible for drafting the manuscript and critical revision of the manuscript for intellectual content. KN was responsible for drafting the manuscript. All authors agree to be accountable for all aspects of the work.

### Conflict of interest statement

The authors declare that the research was conducted in the absence of any commercial or financial relationships that could be construed as a potential conflict of interest.
